# Social Learning in the Real-World: ‘Over-Imitation’ Occurs in Both Children and Adults Unaware of Participation in an Experiment and Independently of Social Interaction

**DOI:** 10.1371/journal.pone.0159920

**Published:** 2016-07-28

**Authors:** Andrew Whiten, Gillian Allan, Siobahn Devlin, Natalie Kseib, Nicola Raw, Nicola McGuigan

**Affiliations:** 1 Centre for Social Learning and Cognitive Evolution, School of Psychology and Neuroscience, University of St Andrews, St Andrews, United Kingdom; 2 Department of Psychology, School of Life Sciences, Heriot Watt University, Edinburgh, United Kingdom; Centre for Coevolution of Biology & Culture, University of Durham, UNITED KINGDOM

## Abstract

The current study avoided the typical laboratory context to determine instead whether over-imitation—the disposition to copy even visibly, causally unnecessary actions—occurs in a real-world context in which participants are unaware of being in an experiment. We disguised a puzzle-box task as an interactive item available to the public within a science engagement zone of Edinburgh Zoo. As a member of the public approached, a confederate acting as a zoo visitor retrieved a reward from the box using a sequence of actions containing both causally relevant and irrelevant elements. Despite the absence of intentional demonstration, or social pressure to copy, a majority of both child and even adult observers included all causally irrelevant actions in their reproduction. This occurred even though causal irrelevance appeared manifest because of the transparency of the puzzle-box. That over-imitation occurred so readily in a naturalistic context, devoid of social interaction and pressure, suggests that humans are opportunistic social learners throughout the lifespan, copying the actions of other individuals even when these actions are not intentionally demonstrated, and their causal significance is not readily apparent. The disposition to copy comprehensively, even when a mere onlooker, likely provides humans, irrespective of their age, with a powerful mechanism to extract maximal information from the social environment.

## Introduction

As a species humans are highly effective social learners, frequently capitalizing on the knowledge and experience of others to acquire useful new behaviors [1, 2]. As a strategy, social rather than individual learning is likely to be extremely useful in many circumstances, circumventing the problem of naïve individuals having to acquire new skills through trial and error, a process which could potentially be costly in terms of time and effort. Such benefits of social learning are readily apparent, but there is also a less obvious pitfall to this process in that the indiscriminate (over)application of social learning may lead observers to acquire suboptimal behavioral variants [3]. This may be particularly true in the domain of tools and artefacts where the function of novel objects is frequently opaque [4], so that although this can be a context in which learning from others is extremely valuable, it leaves observers at the mercy of the competence of the demonstrating individual.

Although it may appear at first glance surprising that human social learning could be so vulnerable to the acquisition of ‘unwanted’ behaviors, a suite of experiments have suggested that not only is the acquisition of suboptimal behavior possible [5–12], it can be extremely resistant to attempts to inhibit it [13–15]. An experimental analogue of such acquisition of suboptimal behaviors has been dubbed ‘over-imitation’ [7] to describe instances where observers copy the superfluous actions performed by a model with such a high level of fidelity that task efficiency is reduced. The typical scenario in over-imitation studies is that an observer watches an adult model demonstrate a series of causally relevant and irrelevant actions on a puzzle-box before the sequence culminates in the retrieval of a reward from inside the box. The most frequent outcome, irrespective of whether the observer is a child or an adult, and even when the irrelevance appears visibly manifest (because the apparatus is made transparent), is one in which the observer reproduces the majority of the actions contained in the demonstration, goal relevant or otherwise, with a high level of fidelity [16]. The tendency of children to over-imitate in such tasks is evident from the age of 3 to 4 years, with subsequent increases in over-imitation occurring later in development, adults reproducing more irrelevant actions than children [16, 17], and older children (six years plus) performing more irrelevant actions than their preschool counterparts [14]. The precision of such copying, by children and adults alike, has been found to extend even to stylistic details in the way in which the individual irrelevant actions were performed [10, 16].

The pervasiveness of over-imitation has been seen as something of a puzzle, and coupled with the finding that the same behavioral tendency occurs in a variety of cultures [14, 18], and has not been found in other primate species [19, 20], has sparked a great deal of experimental and theoretical interest. This has resulted in the generation of many wide-ranging explanatory hypotheses for the phenomenon which encompass social [21], normative [8, 15, 22, 23], pedagogical [24], and informational/causal [7, 25] dimensions of social learning. Under social and normative accounts of over-imitation observers may recognize that the irrelevant actions are not causally necessary, but perform them so as to affiliate with the model by appearing like them [21], or to conform to the social norm of how the object ‘ought’ to be operated [8, 15, 22, 23]. By contrast, informational/causal accounts of over-imitation propose that the observer believes the irrelevant actions to be causally necessary, and so reproduces them, even if their function is somewhat unclear [7, 25]. Despite the differences between these hypotheses, they share an underlying assumption that the apparent pervasiveness of over-imitation means that it is very likely to serve some adaptive function; only the hypothesized underlying motivation or function of the disposition varies.

However, the relevant empirical studies have all been conducted in an experimental ‘laboratory’ context. Although such experimental manipulations of social learning provide invaluable insights into the processes involved, the test contexts have been somewhat unnatural, and may place artificial demands on the observer that would not be present in a real-world context. This may be particularly true of over-imitation, where the participant is asked to view a model perform actions that appear unnecessary for completing the task; a context which has the potential to appear somewhat contrived or at the very least slightly unusual. This atypical context could lead observers, particularly in the case of adults, to interpret the model’s intentional demonstration as an approach to the task that ‘ought to be copied’, rather than revealing anything meaningful about the way in which social learning operates in everyday life. This begs the question of whether observers who were free from such experimental demands would over-imitate, or opt instead to operate more independently and efficiently.

The central aim of the current study was thus to test the real-world occurrence and relevance of over-imitation, in both children and adults, across an age range over which over-imitation has been demonstrated in prior experiments conducted within a laboratory context. This was done by employing a novel methodology in which the task demonstration was detached from the confines of the typical lab set-up, and instead presented in an everyday context in which the participants were unaware that they were taking part in an experiment. In order to do this we capitalized on a resource available to us in the ‘Living Links to Human Evolution Research Centre’, a University of St Andrews primate research center in Edinburgh Zoo. The Living Links Centre showcases live behavioral studies on two primate species and actively supports public engagement with science, with a number of puzzles, games and other interactive items available for visitors to engage with in a ‘Science Exploration Zone’ [26]. We positioned our puzzle-box between these interactive items so that it appeared to be one of the many ‘games’ available to the public rather than an experimental task. From this location the experimenter could easily complete the task in view of an approaching member of the public whilst appearing to be a zoo visitor themselves. This allowed us to eliminate experimental influences whilst providing a realistic vehicle to present a scenario of task completion, including causally unnecessary elements, to the observers.

The novelty of our methodological approach lends itself to a variety of different predictions, each of which varies according to the motivations and/or functions believed to underpin the over-imitative disposition in differently aged observers. One possibility is that the high levels of over-imitation witnessed in both children and adults in conventional lab based studies stem from similar underlying processes, whether these be a susceptibility to social pressure/influence (although the precise social influence may vary with observer age), or the over-employment of a copying process in which the irrelevant actions are viewed as causally necessary. Such commonalities between children and adults would lead us to predict comparable patterns of performance across the two age groups in our naturalistic context; either a blanket reduction in over-imitation due to the alleviation of social pressure, or continued high levels of over-imitation if caused by participants’ assumption that useful information is being gained. Alternatively, children and adults may over-imitate in lab based studies for different reasons, thus potentially generating different patterns of performance when the confines of the traditional experiment are removed in our experiment. It may be that adults, perhaps more cognizant of the redundant nature of the causally irrelevant actions, over-imitate in conventional over-imitation studies due to a sensitivity to the demand characteristics inherent to a lab context (‘it’s what I’m expected to do’), whereas children, who are still relatively inexperienced with both artefacts and cultural conventions, may be copying to learn about how the mysterious novel object works. Such differing functions and their associated motivations would lead to a reduction in over-imitation outside of the laboratory context for adult observers, and potentially older children, but predict continued over-imitation in younger observers more influenced by the content of the naturalistic demonstration.

## Materials and Methods

### Ethics Statement

The study was approved by the Psychology Ethics Committee at St Andrews University (Application #Ps6123). Written informed consent was obtained from the adult participants as well as from the parents of the children who took part.

### Participants

Participants were 93 adults (*M* = 29.6 years, range: 16 to 62 years, SD = 11.2 years), 64 younger children (*M* = 7.5 years, range: 4 to 9 years, SD = 1.4 years), and 64 older children (*M* = 11.6 years, range: 10–15 years, SD = 1.5 years) with an approximately equal number of males and females in each age group. This wide age spread for the children was in part a product of the spontaneous participation inherent in our naturalistic approach. The participants were divided into two groups; a ‘demonstration’ group comprising 100 participants (35 adults and 65 children) and a ‘no-demonstration’ control group comprising 121 participants (58 adults and 63 children). A greater number of adult participants were allocated to the control group than the demonstration group as on occasion zoo visitors, who were earmarked for the demonstration condition, began to interact with the puzzle box before the model was able to commence their actions on the box. Rather than excluding these participants from the study they were allowed to continue to interact with the box and were subsequently included in the control group. Two additional participants were excluded from the study, one who did not wish their data to be recorded, and one who was unable to complete the experiment as English was not their first language.

### Apparatus

**Fig 1:**
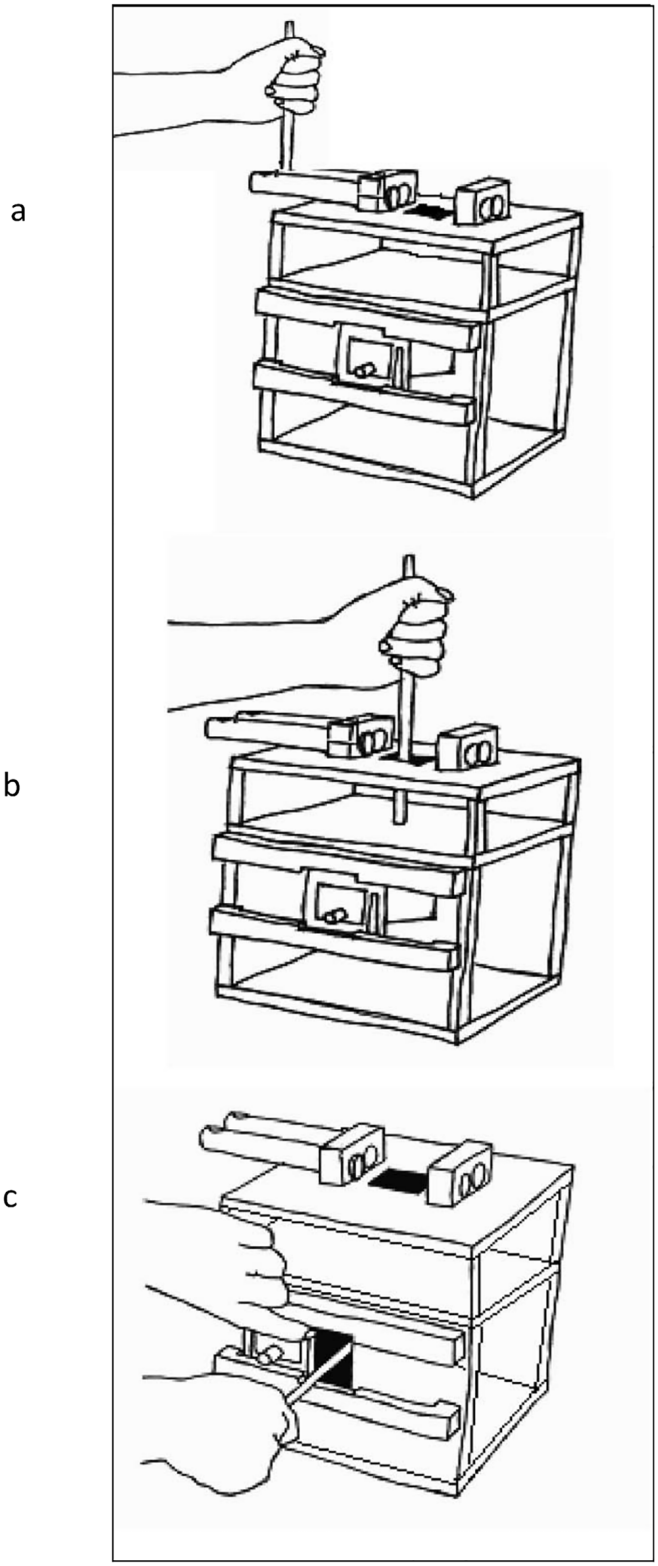
The ‘model’ demonstrates a series of actions, some of which are irrelevant to goal retrieval (removing bolt defence, inserting tool into top hole), and some which are relevant to goal retrieval (removing door defence, inserting tool into lower hole). These actions are illustrated above: (a) dragging bolt; (b) causally irrelevant tool insertion into top hole; (c) causally relevant tool insertion into front hole.

The apparatus used was the same transparent, polycarbonate puzzle box which had been successfully employed with children and adults in previous studies [10,; see Fig 1]. The task demonstration involved the model performing a sequence of causally irrelevant actions towards a bolt ‘defense’ situated on the top of the box before ultimately withdrawing a reward (a magnet-backed laminated note which read ‘*Congratulations*! *Please speak to a researcher and leave this box as you found it*’) from inside the box using a magnet tipped probe. The causally irrelevant elements of the demonstration were always performed first and comprised two stages: 1) the probe was used to slide out the bolt defense in order to uncover a hole on top of the box, and 2) the probe was inserted into the hole and struck multiple times against a false ceiling inside the box. On completion of the irrelevant actions the model performed a two stage causally relevant sequence: 1) a second door defense on the front of the box was manually moved in order to uncover a hole on the face of the box, and 2) the probe was inserted into the hole behind to retrieve the reward from an opaque tunnel. Of interest was whether participants would copy all of the actions irrespective of their causal relevance (i.e., over-imitate), or perform only the actions necessary to obtain the goal.

#### Two-action approach

As well as exploring whether the participants would copy the causally irrelevant actions we were interested in whether stylistic details of the actions would be adopted in this highly naturalistic context. In order to explore this possibility the model removed the bolt defense using one of two different techniques: push the bolts from their back, or drag them from their fronts. If social learning was evident at the stylistic level, the participants would be more likely to perform the observed technique rather than the alternative.

### Procedure

#### ‘Demonstration’ Condition

**Fig 2:**
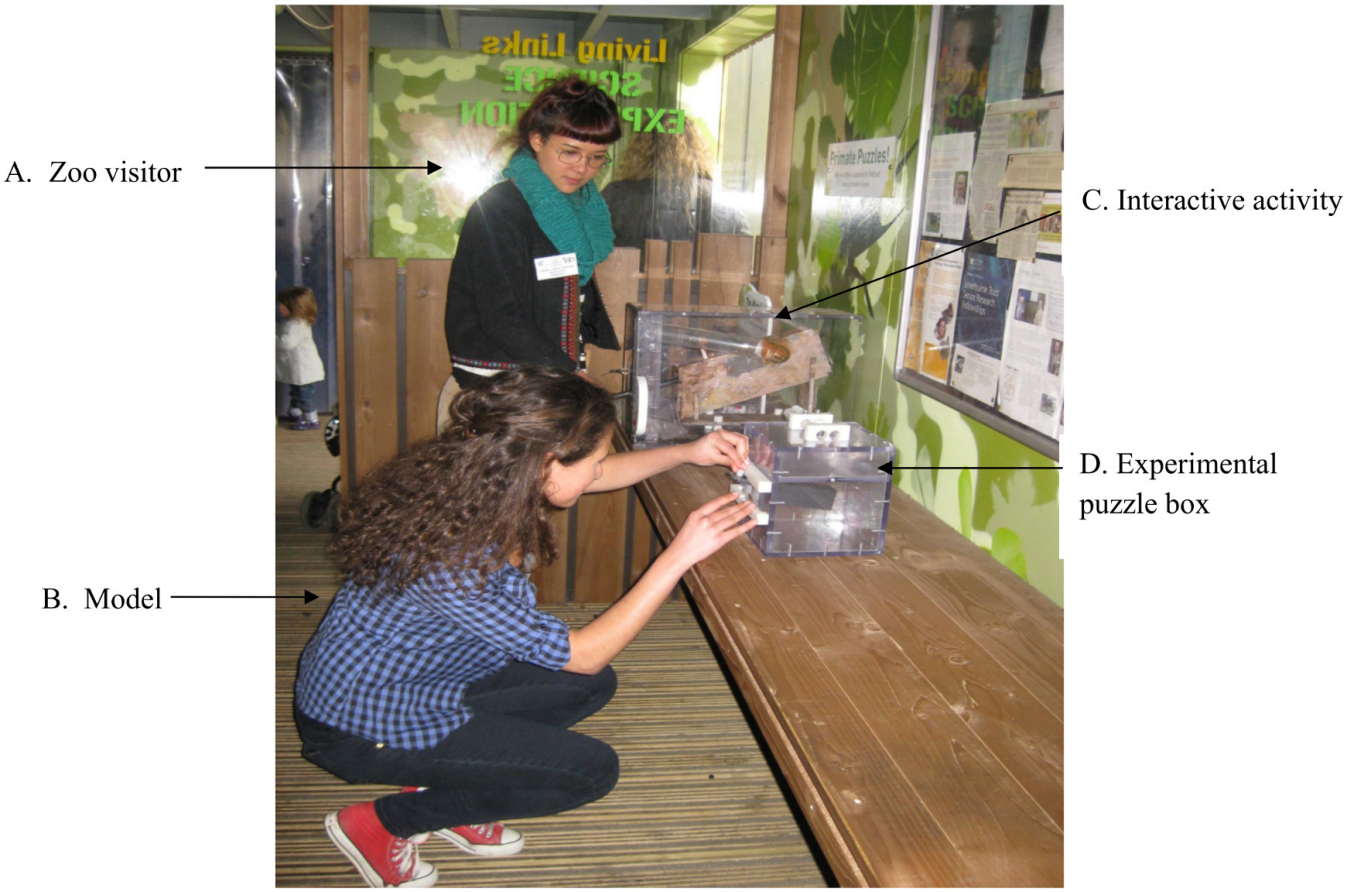
Illustration of the experimental set-up in the Science Exploration Zone of Living Links. Experimenter 2 acting in the role of a zoo visitor (A) observes Experimenter 1 (B) manipulating the experimental puzzle box (D). The puzzle box is situated amongst other interactive activities (C) available for the public to explore.

The overall aim of the ‘demonstration’ condition was to create a context where the task demonstration was viewed by the participants in as naturalistic a manner as possible. In order to achieve this two female experimenters (experimenter 1 who acted as the ‘model’ and experimenter 2 who acted as the ‘onlooker’), both wearing everyday clothes and pretending to be visitors to the zoo, interacted with the items in the vicinity of the puzzle-box as a real zoo visitor, naïve to the puzzle box, approached the ‘games’ area. A visitor was deemed to be naïve to the task if they were known not to have interacted with, or seen any other individual interact, with the puzzle-box previously. This could reliably be known because of experimenters’ continual surveillance of the small Exploration Zone. Once the visitor began to interact with the activity directly adjacent to the puzzle box (positioned <1 meter away to ensure that the demonstration was in clear view of the visitor) experimenter 1 positioned herself in front of the puzzle-box and performed a task demonstration (see Fig 2). To maintain the non-experimental guise of the task, and to motivate the visitor to retrieve the reward, the model laughed when she read the note that she extracted from the box. On completion of the task demonstration the model returned the box to its original state, before moving onto another interactive item out of the visitor’s view as the latter focused on the puzzle box, whilst subtly observing the visitor’s actions on the box. The reinstatement of the puzzle box was the reason that laughter was introduced as a substitute for a reward object to be taken away, because the latter obviously could not have been re-inserted, whereas it was plausible the laminated message was simply popped back in, and the box politely left as it had been found. In order to ensure that the actions performed by the visitor were reliably coded, and that the experimental subject could be confirmed to attend to the task demonstration, a second experimenter equally inconspicuously observed, and mentally noted, the behavior of the participant from behind an interactive video display.

The visitor was allowed to manipulate the puzzle-box for as long as they wished. However, if at any point the visitor successfully extracted the reward from the box, both experimenters approached the visitor and explained that ‘*he/she [the visitor] had taken part in an experiment*’, before asking the visitor whether he/she would be willing to have their data (or their child’s data) recorded. Those participants who indicated that they were willing to have their data included in the study (only one person refused) were asked three questions to ensure that they were unaware that they were taking part in an experiment; (1) ‘*Did you realize that you were taking part in an experiment*?’ (2) ‘*Did you realize you were being watched*?’ (3) ‘*Who did you think this girl [the model] was when you watched her play with the box*?’ (answers consistently confirmed negative responses on the first two questions, and that the model had been believed to be just another zoo visitor). On completion of the experiment both experimenters independently recorded all of the actions performed by the visitor and then agreed on the coding.

#### No-demonstration Control Condition.

Participants in the control condition were zoo visitors who interacted with the box without having viewed either the model, or another zoo visitor, interact with the box previously. The procedure for approaching the visitors and recording their behavior was identical to that used in the demonstration condition with the exception that question 3 was omitted. In both the control and demonstration conditions the experimenters approached, and recorded data from, only those zoo visitors who successfully extracted the reward from the puzzle box. Adopting reward retrieval as our primary inclusion criterion allowed us to meaningfully compare the number of causally irrelevant actions performed in the control and demonstration conditions, a key comparison in determining whether the model’s demonstration influenced the observer’s subsequent approach to the task.

### Coding

#### Irrelevant action score

An ‘irrelevant action’ score was calculated for each participant that ranged from 0 to 2. Participants who reproduced all of the irrelevant actions performed by the model (i.e., removed the bolts and inserted the tool into the top hole) received a score of 2, a partial imitation (i.e., removed the bolts but failed to insert the tool into the top hole) scored 1 point, with those participants who performed no element of the irrelevant sequence scoring 0.

#### Stylistic fidelity score

As well as recording whether or not the participants removed the bolts we also noted the technique they used for doing so. If the participant removed the bolt defense using the same technique as their model they received a score of 1, with those participants who showed no fidelity to the observed technique scoring 0. Instances were a participant performed both the drag and push technique scored 0.5.

#### Inter-rater agreement

The actions performed by each participant were coded independently by the two experimenters. Inter-rater agreement was perfect both for the occurrence of irrelevant actions (Cohen’s κ = 1.0), and the technique used to remove the bolt defense (Cohen’s κ = 1.0); these were highly visible and distinctive alternatives.

## Results

All of the 221 participants who took part in the study indicated that they did not believe themselves to be: a) under the observation of the experimenter(s), or b) taking part in an experiment (two young children required this question to be clarified). In addition all of the participants thought the ‘model’ was a visitor to the zoo. The data from all participants was therefore included in the subsequent analyses (see S1 File).

### Reproduction of causally irrelevant actions

**Fig 3:**
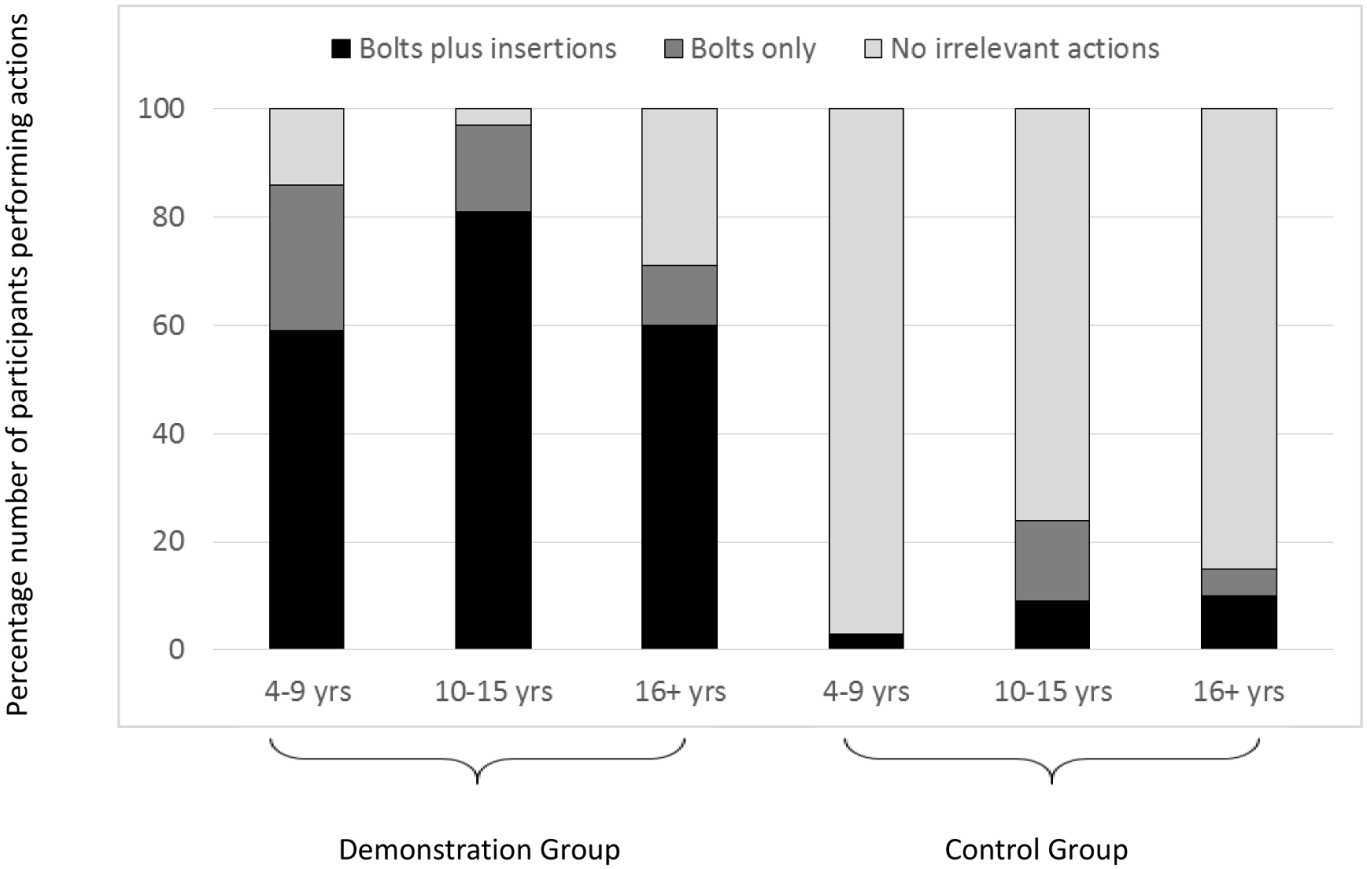
Percentage number of participants in each age group who performed all (bolts plus insertions), part (bolts only), or none of the irrelevant action sequence.

Of initial interest in the analysis was whether the participants would copy the irrelevant actions performed by the ‘model’ when observing these actions in a naturalistic context, and whether any over-imitation witnessed would vary according to the age of the observer. Across the entire sample the majority of the participants who viewed a task demonstration (84 of 100 participants) performed all, or part, of the irrelevant action sequence. In contrast very few irrelevant actions were evident in the ‘No-demonstration control’ condition with only 18 of the 121 control participants performing a complete, or partial, irrelevant sequence (median irrelevant score demonstration condition = 2; median irrelevant score control condition = 0; U(1) = 1694, Z = -10.28, N_1_ = 100, N_2_ = 121, p < .001; see Fig 3). In order to determine whether observer age influenced the level of over-imitation witnessed, the sample was divided into two groups of child participants (younger children: 4–9 years, and older children: 10–15 years), and an adult group (all participants aged over 16 years). Kruskal- Wallis tests conducted on the number of irrelevant actions performed (bolts plus insertions, bolts only, or no irrelevant actions) by each age group (younger children, older children, or adults) revealed that there were no significant age differences in the occurrence of over-imitation in either the demonstration condition (median irrelevant score demonstration condition = 2 at each age), or the control condition (median irrelevant score control condition = 0 at each age). The high levels of over-imitation witnessed within each age group was further evidenced by the significantly greater number of irrelevant actions performed in the demonstration condition, than the control condition, at each age; 4- to 9-year-olds, U(1) = 96.5, Z = -6.17, N_1_ = 34, N_2_ = 30, p < .001; 10- to 15-year-olds, U(1) = 85.5, Z = -6.22, N_1_ = 31, N_2_ = 33, p < .001; adults, U(1) = 428, Z = -5.49, N_1_ = 35, N_2_ = 58, p < .001: see Fig 3.

### Reproduction of stylistic detail: Bolt defense removal

**Fig 4:**
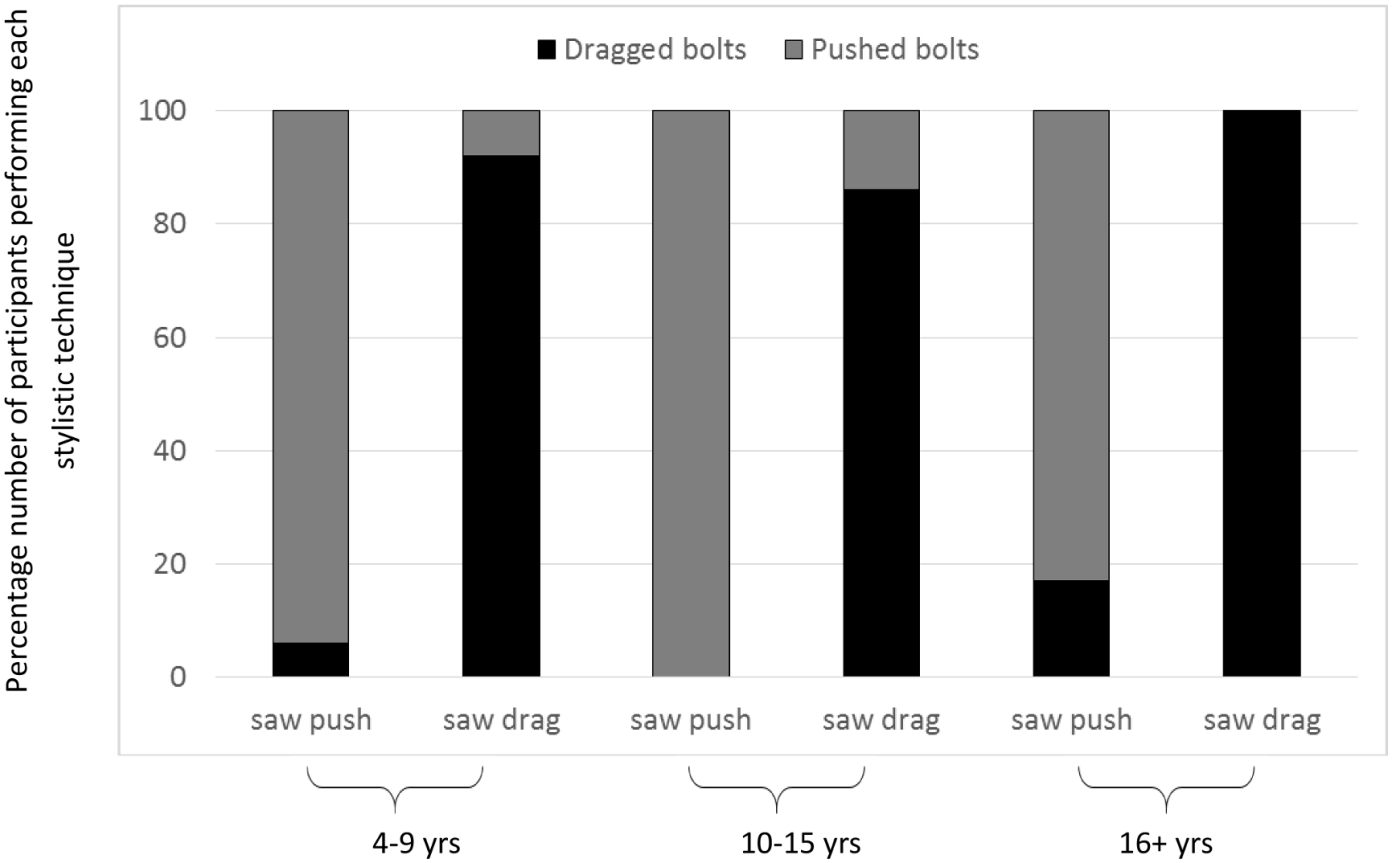
Percentage number of participants in each age group who dragged or pushed the bolts as a function of technique witnessed.

As well as determining whether or not the causally irrelevant actions would be copied in a non-experimental context we were also interested in whether precise details of the technique employed by the model to remove the bolt defense would be faithfully reproduced, and whether any stylistic fidelity witnessed would vary according to the age of the observer. The results show that the fidelity to the technique witnessed was high across the sample, with the majority of the participants who removed the bolt defense in the ‘Demonstration condition’ (N = 84) doing so using the same technique as that performed by their model as opposed to the alternative technique (78 same technique, 6 alternative technique, U(1) = 126, Z = -7.81, N_1_ = 44, N_2_ = 40, p < .001; see Fig 4). The tendency to engage in high fidelity stylistic copying was high irrespective of observer age, with a Kruskal-Wallis test revealing that the tendency to adopt the demonstrated technique did not vary across the three age groups. The high level of stylistic fidelity witnessed within each age group was further evidenced by participants of all ages performing the technique witnessed (median observed technique = 1 at each age) significantly more often than the alternative technique (median alternative technique = 0 at each age), 4–9 years, U(1) = 14.5, Z = -4.55, N_1_ = 16, N_2_ = 13, p < .001; 10–15 years, U(1) = 16.0, Z = -4.70, N_1_ = 16, N_2_ = 14, p < .001; adults, U(1) = 13.0, Z = -4.16, N_1_ = 12, N_2_ = 13, p < .00; see Fig 4.

## Discussion

The central aim of the current study was to eschew the experimental context traditionally used to study over-imitation, in order to determine whether visibly causally irrelevant actions would be copied in a naturalistic context in which the participants were unaware that they were taking part in an experiment, and therefore free from any social pressures inherent to prior lab based studies. In order to achieve such a real-world context our child and adult participants viewed an individual, whom they believed to be another zoo visitor, retrieve a reward from inside a transparent puzzle box, using an action sequence which contained elements that were readily discernible as causally irrelevant. Despite the fact that the model was unknown to the observer, performed the task only once, did not directly ‘teach’ the task to the observer, and physically moved away from the area in which the puzzle box was located before the participant attempted the task, the majority of participants, irrespective of their age, performed the causally irrelevant actions when they themselves attempted the task. That the participants so readily over-imitated in such a ‘stripped back’ learning context demonstrates that merely on-looking can lead an observer to adopt the techniques used by other individuals, even when such techniques appear manifestly inefficient and causally inexplicable. This capacity suggests that humans are opportunistic social learners from a very young age, capitalizing on even fleeting chances to learn fine grained details of action techniques intentionally performed by other individuals on novel artefacts. Perhaps most surprisingly, even the adult participants were subject to this effect despite the causal irrelevance of the actions witnessed being so visible.

The principal question raised by our results is why such a minimal learning context should lead both adults and children alike to abandon their own causal knowledge relevant to the task (displayed in the control condition) and instead adopt the inefficient technique used by the person they had just observed. One important influence which may result in the reproduction of causally irrelevant actions in experimental studies of over-imitation is the social influence of the model. In typical over-imitation studies the model (who is often the experimenter) demonstrates the task directly in front of the observer one or more times, before passing over the task with the instruction “Now it’s your turn”. Of course participants are not invited to imitate, but such experimental contexts likely contain a number of social influences which may impact on the likelihood that the observers will over-imitate. First, these influences may elicit a motivation on the part of the observer to affiliate with the model by acting like them; second, they may place social pressure on the observer to conform to performing the task in the same way as the model; or third, they may provide an opportunity for the observer to engage in a shared experience with the model [13, 21, 27, 28](for a review see [29]). In our naturalistic context all such potential social demands were essentially absent, suggesting that such social influences were unlikely to be the motivation behind the over-imitation witnessed. The lack of a direct face-to-face demonstration by the model also rules out a fourth social influence, that the occurrence of over-imitation was dependent on pedagogical cues [30, 31], as no such cues were present in the display witnessed. The high fidelity copying nevertheless witnessed across our sample suggests that other age independent motivations may be responsible for eliciting over-imitation in such a context. To be clear, we are not suggesting that such direct social influences never play a role in motivating over-imitation; to the contrary, a range of studies show they can [6, 23, 27]. But within a naturalistic context, merely viewing another individual’s task solution can elicit a ‘copy-all’ strategy.

If the social theories outlined above cannot readily explain the high fidelity copying within our naturalistic context then perhaps informational/causal theories may provide greater insight into the behavior of the participants. Causal theories of over-imitation share in common that the causally irrelevant actions are included in the participant’s reproduction as these actions are assumed to be necessary to achieve the goal (even if their exact purpose remains unspecified). In one such theory, “copy-all, refine later”, it is proposed that children who over-imitate are displaying an adaptive disposition to initially copy all of the model’s actions, then later refine their approach as necessary on the basis of experience, thus weeding out any occasional mistaken causal links [25, 32]. A twist on this theory, dubbed the “Automatic Coding Hypothesis”, proposes that children automatically encode all actions intentionally performed by a model as causally necessary [7, 13]. The automatic coding hypothesis predicts extreme fidelity in the copying of irrelevant actions, leaving little room for the flexible deployment of copying behavior. The high levels of over-imitation witnessed in the present study are consistent with such an apparently ‘indiscriminate copying’ account of over-imitation, and perhaps surprisingly, suggest that a relatively blanket copying process is not restricted to early childhood, where one may anticipate such a strategy to be most useful, and instead continues into adulthood. However, the performance of the minority of participants in each age group who omitted all of the causally irrelevant actions indicates that high fidelity copying is not as consistent as the theory of automatic coding would predict (c.f. [14], [33–36]. This suggests that although the levels of over-imitation we observed are broadly consistent with an automatic coding account, it is likely that such a mechanism, if it were employed, involves a greater degree of flexibility than that proposed by Lyons and colleagues.

However, a final explanation to be considered is that although direct social interaction is not necessary for the strong over-imitation effects we observed, participants may nevertheless have perceived the actor’s performance as prescriptively normative; that is, an approach that one *ought* to take, because it is a cultural norm. This may also be regarded as a socially-based explanation competing with informational/causal explanations, although a more indirect one if it can occur without direct social interaction. Evidence that children may over-imitate because of prescriptive normativity comes from recent studies using a ‘protest paradigm’. These have shown that preschool children who initially viewed an adult model perform an action sequence containing causally irrelevant actions frequently protested when a puppet subsequently omitted the causally irrelevant actions from their reproduction [8, 15, 22, 23]. Finding that this may occur even when the puppet successfully completes the task and obtains the reward, Kenward [8] argues this rejects the automatic causal encoding hypothesis in favor of an effect whereby children instead tend to view all adult actions as prescriptively normative. Could this explain our results? Reasons to think not refer to two main potential bases for the activation of prescriptive normativity. One derives from descriptive normativity, where one observes that a form of action is common in a community; a substantial corpus of intriguing studies in the broader influence literature show that perceiving what is descriptively normative in a group leads experimental participants to assume and act under the influence of prescriptive normativity [37]. This is surely not relevant in our experiment since participants saw only a single model act on the puzzle-box a single time. The second basis is where the model offers cues that actions are prescriptively normative, which does not require a group effect and can in principle be elicited by a single model. For example, when preschool children in one experiment were presented with a sequence of actions which were verbally framed as conventional (‘rituals’), imitative fidelity increased over that witnessed with instrumental framing (and this occurred particularly when the verbal frame was accompanied by the other indicator of social conventionality noted above, multiple synchronous models [38]. Such effects also occurred in the protest-paradigm over-imitation studies cited above, with a solo model. However, these appeared replete with cues to prescriptive normativity, which may be broadly thought of as pedagogical. Thus in Kenward’s study [8], in a warm up session children were already encouraged to watch what an adult did and then do it, and help correct the mistakes of a puppet doing the same; then in the experiment proper, “the demonstrator first instructed the child and puppet to watch” (p. 200), a cue that what followed is what the child should note is what one ought to do.

Such cues were explicitly avoided in our experimental configuration, so that neither of the two bases for eliciting prescriptively normative responses outlined above were in play, implying that the over-imitation we observed occurred because of participants encoding of the assumed informational and causal content of the irrelevant actions. However, we note that like many concepts and terms in our field, ‘normativity’ has been interpreted by some authors to cover a very broad spectrum, including not only being influenced by the most obvious socially-related case of rituals and other cultural conventions, but also by informational and causal instances, insofar as one ‘should’ do whatever best achieves one’s instrumental goals, and the norms of one’s community are a good guide to this. As Kenward [8] puts it, “In this case, the relevant normative domain is that of instrumental rationality, according to which, all else being equal, an agent should perform an action that leads to the agent’s goal” (p. 197). However, this is a very different sense of ‘normative’ to that related to cultural conventions, and in the case of our experiment, given that the participant has no additional evidence that what the model does is normative in the sense of being common in the community, arguably amounts to no more than a rule akin to ‘copy what another person does because it is likely a good guide to what will work in this case’, as suggested in our original ‘copy-all, refine later’ hypothesis [32].

Whatever the outcome of such ongoing debates about the underlying explanations for over-imitation, the novelty of the current findings lies in demonstrating that over-imitation may occur in both children and adults even in contexts of minimal social ‘demand’, for in our study the usual ‘lab’ experimental context was replaced by one where the participant did not know at the time they were in an experiment, and they were left by themselves to perform whatever actions they wished. These results show that although evidence shows that in some contexts direct social interaction, including pedagogy, can enhance over-imitation, they are not essential causal factors (c.f. [23]). A capacity to rapidly acquire new behaviors outside of a social or pedagogical context likely serves an important function. Although pedagogy can be important in the transmission of cultural behavior from one individual to another, particularly from adult to child, without a capacity to capitalize on the information that we merely ‘eavesdrop’ on, many opportunities for the rapid acquisition of novel skills and social conventions would be missed. Importantly, the current results suggest that this capacity for fast acquisition is not restricted to early childhood, with humans continuing to engage in a ‘copy-all, refine later’ strategy up to, and throughout, adulthood. Thus rather than being an artefact of a contrived experimental context, the occurrence of over-imitation in the real world, as demonstrated in our study, likely maximizes the power of the human capacity to obtain all the practical and conventional skills that are foundational to human culture.

## Supporting Information

S1 File DataFile. Data set that includes the over-imitation scores of both the child and adult participants.
(SAV)
